# Causes, Weaknesses, Strengths, and Lessons Learned from Incident Management in Bandar Abbas, Iran, 2025

**DOI:** 10.34172/aim.34641

**Published:** 2025-09-01

**Authors:** Milad Ahmadi Marzaleh, Mahmoudreza Peyravi, Fatemeh Nematollahi

**Affiliations:** ^1^Department of Health in Disasters and Emergencies, Health Human Resources Research Center, School of Health Management and Information Sciences, Shiraz University of Medical Sciences, Shiraz, Iran; ^2^Student Research Committee, Shiraz University of Medical Sciences, Shiraz, Iran

## Dear Editor,

**Figure 1:**
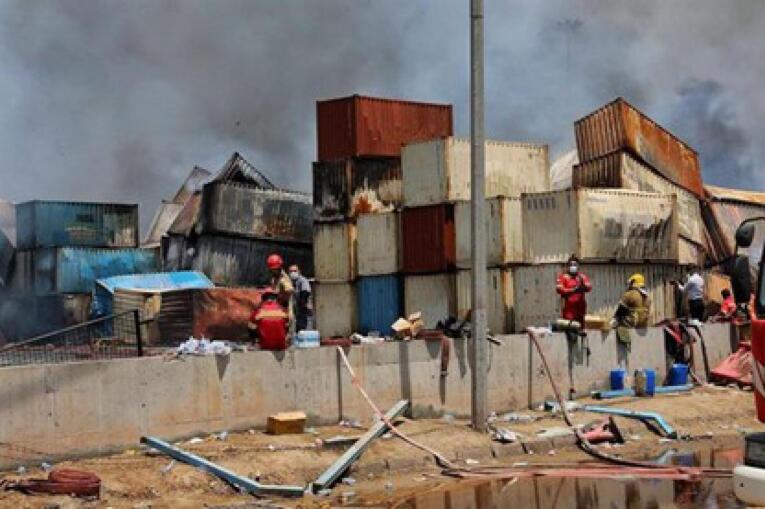
After the Explosion (Creator: Iranian Red Crescent | Credit: via Reuters).

 With industrial development, accidents caused by industrial activities have also increased, sometimes endangering the industry or its employees. Most accidents in industry are usually due to the leakage of a flammable or toxic substance into the environment, which can occur due to ruptured lines or defects in connections, which causes an explosion.^1^ Explosion injuries are increasingly common, and most victims of this injury do not die immediately; also, most survivors suffer from severe injuries.^2^ Explosions can destroy healthcare infrastructure and lead to increased demand for medical care, and, in some cases, the high number of casualties from disasters exceeds the capacity of the health system to provide the necessary services. A previous explosion was the massive one on August 4, 2020, in Beirut, the capital of Lebanon, when a warehouse containing 2,750 tons of ammonium nitrate exploded, injuring more than 6,500 people, killing 220, and leaving 30,000 people homeless.^3^ Therefore, this study aimed to examine the causes, strengths, weaknesses, and lessons learned from the management of the explosion incident in Bandar Abbas, Iran, in 2025 (Figure 1).

 Regarding the explosion at Shahid Rajaee Port in the Hormozgan Province, Iran, potentially due to mislabeled or improperly stored hazardous materials such as flammable liquids or chemicals between inside containers and inadequate safety protocols and insufficient infrastructure on April 26, 2025, at 12:09 PM, more than 1,240 people were injured, and 57 people died. In addition to the fire and blast wave, toxic gases also polluted the air in Bandar Abbas.

 The measures taken in response to this incident included holding crisis management meetings in the Hormozgan governorate, taking immediate measures by the Red Crescent, such as sending 25 operational teams, 55 support teams, as well as 5 helicopters to the Hormozgan province to control the fire with fire extinguishing powders. Also, 15 pre-hospital ambulances, one ambulance bus, one sea ambulance, and 8 ambulances from other provinces (such as Fars) were sent to the scene of the incident. Due to toxic gas emissions, residents were warned not to leave their homes as much as possible or to use N95 masks if necessary.

 The strengths of incident management include:

1. Calling for the presence of the Ministers of Interior and Roads and Urban Development at the scene of the incident for decisive management and better command of the incident;
2. Dispatching rapid response teams and Red Crescent assessors to the scene of the incident;
3. Dispatching pre-hospital emergency teams to the scene of the incident to transport the injured;
4. Announcing blood transfusion organizations in 7 provinces to transfer blood to Bandar Abbas hospitals;
5. Calling for cooperation of military helicopters for firefighting operations;
6. Holding an urgent emergency response meeting to the incident;
7. Announcing readiness of 50 operational and medical teams in the provinces of Kerman, Yazd, Bushehr, Sistan and Baluchestan, Fars, and Isfahan;
8. Dispatching 50 operational teams to the scene of the incident;
9. Dispatching 5 Red Crescent Society helicopters for relief, support, and operations;
10. Enhanced communication systems to ensure timely and coordinated responses

Weaknesses and challenges of incident management include:

1. Preparation of people for specific man-made events such as explosions;
2. Lack of proper training about explosive events and related reactions;
3. Lack of dissemination in cyberspace management of rumors and arousing people’s emotions
4. Creation of contradictory information about the explosion by news reports and cyberspace.

Ultimately, the lessons learned are:

1. Improving inherent safety protocols in ports;
2. Performing risk assessments in short time frames;
3. Improving public knowledge and awareness regarding explosive incidents;
4. Training employees to prevent possible explosions;
5. Managing emotions and individual resilience;
6. Collaborating with crisis management organizations such as the Red Crescent, pre-hospital emergency services, and the governorate;
7. Increasing the resilience of communities when faced with disasters.

## Conclusion

 As a result of industrial progress in the world and Iran and the possibility of increasing explosive accidents, the need for the presence of experienced and knowledgeable crisis managers helps to manage these accidents. Also, writing protocols in coordinating organizations such as the police, supporting organizations such as the Red Crescent and Emergency, and the Crisis Management Organization can play a fundamental role in carrying out coherent and correct management to reduce the consequences of the accident. Thus, such accidents can be prevented in the first place and, if they occur, the damage to humans, equipment, and environment can be reduced.
